# Underlying neurological mechanisms associated with symptomatic convergence insufficiency

**DOI:** 10.1038/s41598-021-86171-9

**Published:** 2021-03-22

**Authors:** Tara L. Alvarez, Mitchell Scheiman, Cristian Morales, Suril Gohel, Ayushi Sangoi, Elio M. Santos, Chang Yaramothu, John Vito d’Antonio-Bertagnolli, Xiaobo Li, Bharat B. Biswal

**Affiliations:** 1 Biomedical Engineering, New Jersey Institute of Technology, Newark, NJ USA; 2 Pennsylvania College of Optometry, Salus University, Philadelphia, PA USA; 3 Department of Health Informatics, Rutgers University School of Health Professions, Newark, NJ USA

**Keywords:** Neuroscience, Oculomotor system, Cerebellum, Cortex

## Abstract

Convergence insufficiency (CI) is the most common binocular vision problem, associated with blurred/double vision, headaches, and sore eyes that are exacerbated when doing prolonged near work, such as reading. The Convergence Insufficiency Neuro-mechanism Adult Population Study (NCT03593031) investigates the mechanistic neural differences between 50 binocularly normal controls (BNC) and 50 symptomatic CI participants by examining the fast and slow fusional disparity vergence systems. The fast fusional system is preprogrammed and is assessed with convergence peak velocity. The slow fusional system optimizes vergence effort and is assessed by measuring the phoria adaptation magnitude and rate. For the fast fusional system, significant differences are observed between the BNC and CI groups for convergence peak velocity, final position amplitude, and functional imaging activity within the secondary visual cortex, right cuneus, and oculomotor vermis. For the slow fusional system, the phoria adaptation magnitude and rate, and the medial cuneus functional activity, are significantly different between the groups. Significant correlations are observed between vergence peak velocity and right cuneus functional activity (*p* = 0.002) and the rate of phoria adaptation and medial cuneus functional activity (*p* = 0.02). These results map the brain-behavior of vergence. Future therapeutic interventions may consider implementing procedures that increase cuneus activity for this debilitating disorder.

## Introduction

Convergence insufficiency (CI) is the most common binocular vision disorder across the age spectrum, with an estimated prevalence of 4.2% to 17.6% in children^1–5^, and 3.4% to 7.7% in adults^6–9^. This condition responds well to therapeutic intervention^10^; hence it presents a unique opportunity to better understand the neuroscience of neuroplasticity. This common condition has a negative impact on a person’s quality of life because its associated symptoms include sore eyes, headaches, blurred vision, double vision, and loss of concentration^11,12^. These symptoms are exacerbated when doing prolonged near work, such as reading, computer usage, or using any electronic device such as a smartphone, tablet, or a virtual reality headset^11,12^. In the past 15 years, the results of many randomized clinical trials^13–24^ have demonstrated the effectiveness of various interventions for the treatment of CI. However, even the most effective treatment (office-based vision therapy) is only successful about 75% of the time and requires a minimum of 25 h of treatment (typically a combination of office and home-based therapy)^13,22^. Thus, there is a need for novel evidence-based protocols to optimize therapeutic interventions. Optimized treatment may lead to improved functional outcomes in shorter periods of time, as well as positive benefits related to cost-effectiveness and healthcare resource allocation. Some of the ways to accomplish these objectives include the following: (1) understand how brain-behavior function differs between binocularly normal controls (BNC) and CI participants, (2) identify what elements of brain function change during rehabilitation, and (3) determine how brain activity is correlated to visual behavior function post-therapeutic intervention.

The purpose of this study was to investigate the underlying neural mechanistic differences between BNC and symptomatic CI participants by assessing baseline measurements. To do so, we designed two experiments using a multimodal approach integrating functional brain imaging, behavioral assessments (eye movement and phoria adaptation), and a comprehensive vision examination that included clinical signs and symptoms. The experimental design compared the fast and slow fusional disparity vergence systems in BNC versus CI participants. Fast fusional vergence is the process by which the eyes quickly track a target moving in depth, analogous to a baseball batter tracking a fastball^25^. Slow fusional vergence is the process by which the brain optimizes sustained vision (used during reading) to either near or far visual space^26^. Once the underlying neural substrate difference(s) are understood then therapeutics can be evaluated to understand how the intervention modifies the underlying functional activity of the brain to ultimately reduce vision symptoms. Understanding the neural mechanism of CI is a critical first step towards the optimization of therapeutic interventions via improving success rates along with potentially decreasing treatment duration and cost.

## Results

### Clinical signs and symptoms

We enrolled 50 BNC (21.8 ± 3.3 years old, 30% female), and 50 symptomatic CI (20.9 ± 3.6 years old, 50% female) participants. There were no significant differences in age, refractive error, or stereopsis between the BNC and CI groups *p* > 0.1^27^. The details of race, ethnicity, and refraction are available in a previously published paper and were not substantially different between the BNC and CI groups^27^. The mean near point of convergence for the CI group was 10.4 ± 3.5 cm, which was significantly more receded compared to the BNC group of 3.8 ± 1.2 cm [t(98) = 12.5, *p* < 0.0001]. For positive fusional vergence, the CI group mean was 12.5 ± 4.0∆, which was significantly less than the BNC group mean of 27.8 ± 8.4∆ [t(98) = 13.4, *p* < 0.0001]. The CI group near horizontal dissociated phoria (6.9 ± 3.2∆) was significantly more exophoric compared to the BNC group (2.0 ± 2.1∆) [t(98) = 8.91, *p* < 0.0001]. The far horizontal phoria levels were not significantly different between the CI and BNC groups (CI group = 0.5 ± 2.1∆ exo versus BNC group = 0.11 ± 0.54∆) [t(98) = 1.41, *p* = 0.2]. When comparing the difference in the near to the far horizontal phoria, the CI group had a significantly greater amount of exophoria at near versus far (6.3 ± 2.4∆) compared to the BNC group (1.9 ± 2.2∆) [t(98) = 9.6, *p* < 0.0001]. This shows that the AC/A ratio was lower in the CI group compared to the BNC group. The CI participants were significantly more symptomatic with a score of 34.5 ± 7.6 points on the CISS compared to the BNC CISS score of 8.2 ± 5.3 points [t(98) = 20.0, p < 0.001].

### Fast fusional vergence experimental results

**Figure 1:**
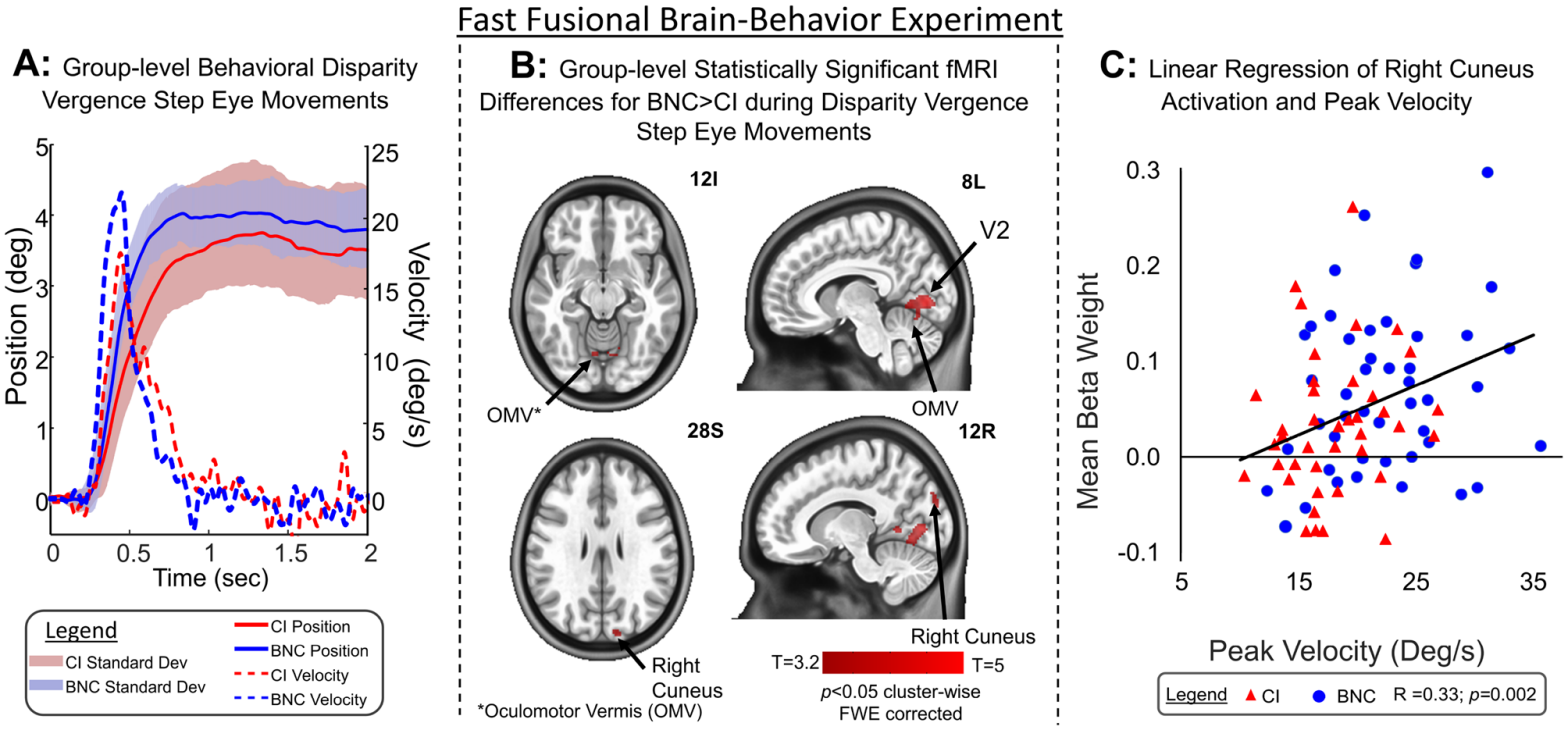
(**A**) Averaged vergence eye movement position (°) as a function of time (s) from BNC (solid blue line) and CI (solid red line) participants with ± one standard deviation (BNC blue shaded and CI red shaded) with the averaged peak velocity (°/s) as a function of time (s) from BNC (dashed blue line) and CI (dashed red line) participants. (**B**) Two-sample unpaired t-test of functional activity differences between BNC and CI groups during the vergence jump step fast fusional experiment showing V2, OMV, and right cuneus are significantly different between groups. (**C**) Linear regression of mean beta weight from right cuneus ROI using a 5 mm radius sphere as a function of peak velocity for BNC (blue circle) and CI (red triangle) participants. Linear regression (solid black line) analysis shows Pearson’s correlation coefficient of r = 0.33, *p* = 0.002 between right cuneus functional activity and vergence peak velocity.

Figure 1A shows the group-level average position trace (solid blue line in the unit of as a function of time in the unit of seconds) of the BNC group stimulated by a 4° symmetrical disparity jump step with plus and minus one standard deviation (shaded light blue) and the average velocity trace (dashed blue line in the unit of °/s as a function of time in the unit of seconds). The CI group average position trace (solid red line) with plus and minus one standard deviation (shaded light red) and velocity trace (dashed red line) were plotted with the BNC results to compare the averaged convergence responses between groups. There were 1 BNC and 3 CI participants who blinked and/or saccaded so excessively during the transient portion of the vergence response so that no meaningful data could be analyzed. Those participants were not included within this group-level analysis. The peak velocity for the BNC group was 21.9 ± 5.6°/s which was significantly greater than the CI group’s peak velocity of 17.7 ± 4.0°/s [t(94) = 4.22*, p* < 0.0001]. The final amplitude for the BNC group was 3.7 ± 0.36°. The CI group was significantly less than the BNC group measured at 3.4 ± 0.62° [t(94) = 3.0, *p* < 0.005]. The latency of the 4° convergence responses was not significantly different between the CI (223 ± 31 ms) and BNC (218 ± 21 ms) groups [t(94) = 0.9, *p* = 0.4].

For the fast fusional vergence step functional MRI experiment, 1 BNC participant and 6 CI participants were removed from the group-level analysis due to motion artifacts that failed the criteria described within the methodology. Averaged functional activity using a one-sample t-test with the experimental design for the BNC and CI groups were shown in the Supplemental Materials. The following neural substrates were identified using one-sample t-tests as shown in the Supplemental Material for both visual tasks and both groups of participants: frontal eye fields (FEF), parietal eye fields (PEF), supplementary eye field (SEF), bilateral and medial cuneus, oculomotor vermis (OMV), and visual cortex^28–32^. The group-level two-sample unpaired t-test results for the fast fusional experiment comparing sustained gaze fixation to a block of 8 vergence eye movements sought to determine which regions of interest (ROIs) were significantly greater in the BNC versus CI groups. Results were shown in Fig. 1B, corrected for multiple comparisons using family-wise error (FWE) cluster-wise correction. The following ROIs were significantly different between the BNC and CI groups: right cuneus, secondary visual cortex (V2), and left and right dorsal vermal region VI, which has been defined as the oculomotor vermis (OMV)^33^. The MNI coordinates which had the greatest T value and the uncorrected *p*-value per ROI were the following: right cuneus (MNI: 12x, − 80y, 28z) [t(74.3) = 3.95, *p* < 0.0001]; V2 (MNI: − 8x, − 76y, 0z) [t(85.5) = 5.20, *p* < 0.0001]; right vermal region VI (MNI: 6x, − 70y, − 16z) [(87.3) = 3.53, *p* < 0.0001]; and left vermal region VI (MNI: − 8x, − 68y, − 12z) [t(88.3) = 3.68, *p* < 0.001]. Using an unpaired t-test, the mean beta weight from the 5 mm radius sphere within the right cuneus for the fast fusional experiment were significantly greater in the BNC group (mean with standard deviation = 0.07 ± 0.08) compared to the CI group (0.03 ± 0.08) [t(85) = 2.33; *p* = 0.02].

The ROI-based and voxel-based correlation analyses between the functional activity beta weights and vergence eye movement peak velocity were similar. Only the right cuneus functional activity was significantly correlated to the peak velocity of the 4° vergence step responses. Figure 1C plots the mean beta weight time series within a 5 mm radius sphere from the MNI coordinate 12x, − 90y, 28z that was identified as the most significantly different location within the right cuneus as a function of the peak vergence velocity from 4° step stimuli per participant. The linear regression analysis of BNC (blue circle) and CI (red triangle) participants results in a Pearson’s correlation coefficient of r = 0.33 (*p* < 0.002) from the 5 mm sphere, supporting that the functional activity of the right cuneus was correlated to the maximum speed of vergence step responses. The final amplitude of the vergence step was not significantly correlated to the right cuneus activation *p* > 0.1. The beta weight means from two other spheres of 3 mm and 7 mm radius were also computed. The Pearson’s correlation values between the mean beta weights and the peak velocity of the 4° vergence step responses were r = 0.29 (*p* = 0.006) and r = 0.35 (*p* < 0.001) for the 3 mm and 7 mm radius spheres, respectively.

### Slow fusional vergence experimental results

**Figure 2:**
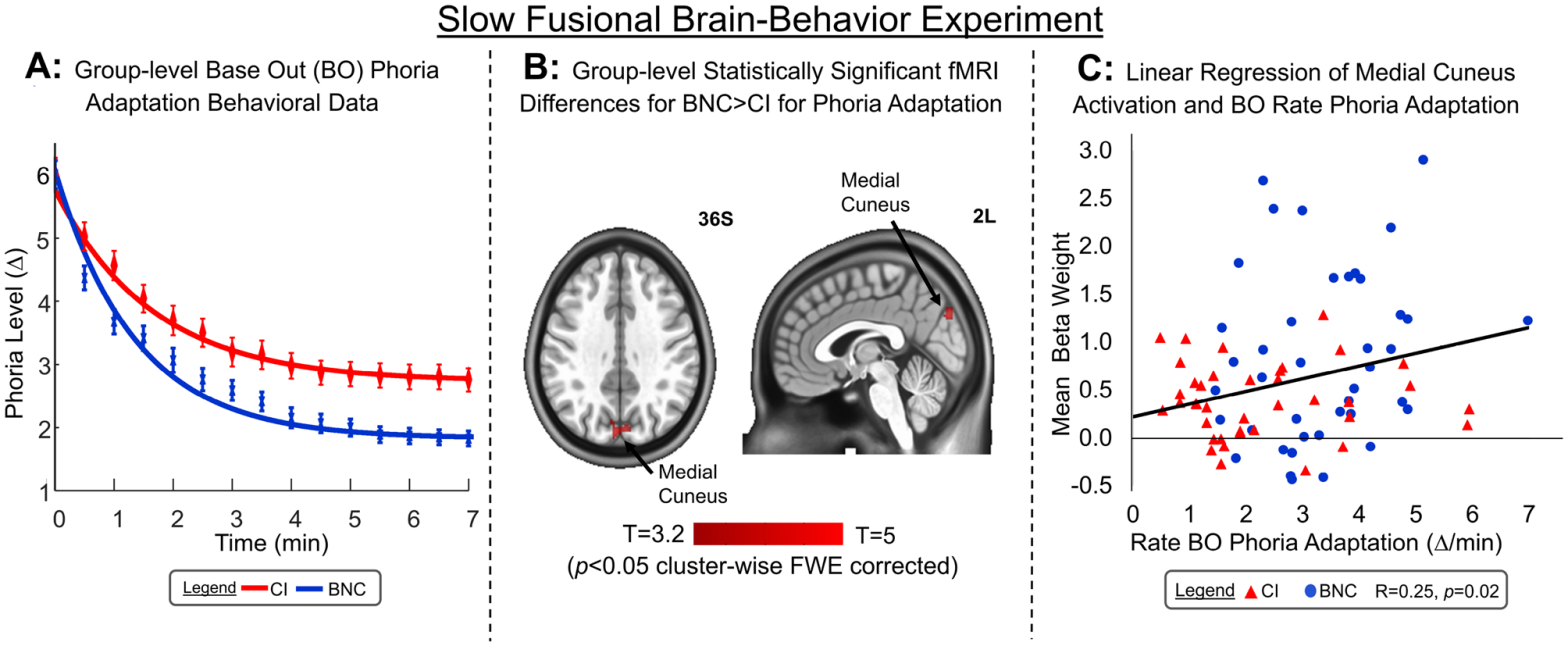
(**A**) Group-level averaged phoria adaptation results of phoria (∆) as a function of time (min) using a 6∆ base-out (BO) prism for BNC (blue symbols) and CI (red symbols) groups ± 1 standard error of the mean. Exponential decay fit of BNC group (solid blue line) and CI group (solid red line). (**B**) Two-sample unpaired t-test of functional activity differences between BNC and CI groups during the phoria adaptation slow fusional experiment showing medial cuneus was significantly different between groups. (**C**) Linear regression of mean beta weight from medial cuneus ROI using a 5 mm radius sphere as a function of the rate of phoria adaptation (∆/min) to 6∆ BO prism for BNC (blue circle) and CI (red triangle) participants. Linear regression (solid black line) analysis shows the Pearson’s correlation coefficient of r = 0.25, *p* < 0.02 between medial cuneus functional activity and rate of phoria adaptation.

The averaged phoria adaptation results stimulated using a 6∆ base-out (BO) prism for BNC (blue), and CI (red) participants were plotted with an exponential decay fit (solid lines) in Fig. 2A. Phoria adaptation rates and magnitudes that were two times greater than the average for each group were removed as outliers. For the BNC group, the averaged magnitude of phoria adaptation was 4.2∆ with a standard error of the mean (used to compare with other prior literature) of 0.2∆ that was significantly greater than the CI group’s average of 3.2 ± 0.2∆ [t(83) = 3.53, *p* < 0.0001]. The rate of phoria adaptation between the groups was 3.4 ± 0.2 ∆/min for the BNC and 2.5 ± 0.3 ∆/min for the CI groups, which was significantly different [t(83) = 2.46, *p* = 0.016].

From the slow fusional functional MRI phoria adaptation experiment, 2 BNC participants and 6 CI participants were removed from the group-level analysis due to motion artifacts defined within the methodology. The Supplementary Material contains the significant functional activity spatial maps of the BNC and CI group-level results for the slow fusional fMRI experimental task. Figure 2B was the 2-sample unpaired t-test of the BNC and CI participants showing which regions were significantly different between the groups, corrected for multiple comparisons using FWE cluster-wise correction. The region that was significantly different between the groups was the medial cuneus. The MNI coordinate that was the most significantly different between the groups was − 2x, − 82y, and 36z; [t(83.9) = 4.37, *p* < 0.0001]. Figure 2C was the mean functional activity beta weight from a 5 mm radius sphere centered at the MNI coordinate with the peak difference as a function of the rate of BO phoria adaptation. Using an unpaired t-test, the mean beta weight from the 5 mm radius sphere within the medial cuneus of the slow fusional experiment were significantly greater in the BNC group (mean with standard deviation = 3.28 ± 1.25) compared to the CI group (2.25 ± 1.42) [t(78) = 3.45; *p* < 0.001]. The BNC participants were plotted with blue circles, and the CI participants were plotted with red triangles. The linear regression analysis plotted as a solid black line reports a significant correlation between the mean beta weight activity in the medial cuneus using a 5 mm radius sphere and the rate of BO phoria adaptation with a Pearson’s correlation coefficient of r = 0.25 (*p* < 0.02). The mean beta weight from two other size spheres were calculated: 3 mm and 7 mm radius. The Pearson’s correlation coefficient between the mean beta weight and the rate of BO phoria adaptation was r = 0.25 (*p* < 0.02) and r = 0.26 (*p* < 0.02) for the 3 mm and 7 mm radius spheres, respectively. The magnitude of phoria adaptation was not significantly correlated to the medial cuneus activation *p* > 0.1.

## Discussion

This study reports the following: neural substrates that are functionally active during the fast and slow vergence systems for BNC and CI groups; objective evidence of dysfunction in the CI participants for behavior and functional activity in comparison to BNC results; and brain-behavior correlations providing support regarding which neural substrates correspond to behavioral differences observed between BNC and CI participants. Two significant brain-behavior correlations were observed for the fast and slow fusional vergence eye movement systems, respectively.

The fast-fusional system is responsible for fusing binocular stimuli that are rapidly changing in the environment, such as alternately looking between two fingers centered along a person’s midline. Our results demonstrate that symmetrical vergence jump steps stimulated within a functional MRI experiment evoke functional activity within the visual cortex, cuneus, PEF, FEF, SEF, and cerebellar regions. Vergence eye movements are significantly slower, and functional activity within V2, OMV, and the right cuneus is significantly reduced, in CI participants compared to BNC. Correlation analyses support that functional activity within the right cuneus is correlated to the peak velocity stimulated by symmetrical disparity vergence jump steps.

The slow-fusional system is responsible for optimizing the brain’s effort after prolonged viewing, such as reading a book or playing a video game for an extended period. Our results demonstrate that phoria adaptation stimulates the visual cortex, cuneus, PEF, FEF, and cerebellar regions. CI participants had significantly reduced magnitude and rate of phoria adaptation to a 6∆ BO prism as well as reduced functional activity within the medial cuneus compared to the BNC group. Correlation analyses demonstrate that the functional activity within the medial cuneus is significantly correlated to the rate of base-out phoria adaptation. The two significant correlations demonstrate that different areas of the cuneus are activated for the fast and slow fusional disparity systems.

### Comparison to other behavioral literature

Our group completed a pilot study demonstrating that young adult CI participants had significantly slower peak velocity compared to BNC^28^. Slower peak velocity in CI participants has also been observed by others in the young adult population^34^, the pediatric population^35^, concussed pediatric with CI population^36^, and mild traumatic brain injury with CI participants^37^. The novelty of this current work is that it is the first to demonstrate a correlation between vergence peak velocity and the right cuneus functional activity.

The slow fusional vergence system is assessed via phoria adaptation. Several studies provide evidence that phoria adaptation is reduced in terms of the magnitude and the rate of adaptation in CI participants compared to controls^26,38,39^. Phoria adaptation in CI participants improves post vision therapy^40^. The novelty of this research is that a significant correlation is observed between functional activation within the medial cuneus and the rate of base-out phoria adaptation.

### Cuneus involvement in visual tasks

Reviews on the neural circuit of disparity vergence concentrating on the electrophysiology studies have not yet studied the cuneus^41,42^. Prior functional imaging studies in BNC do report functional activity of the cuneus during vergence jump step experiments^29^, during stimulation of stereopsis^43^, and for optokinetic eye movements^44^. Exogenous attention^45^ and visual attention^46^ also stimulate the cuneus within functional imaging experiments. Interestingly, epidemiology studies report attention deficit hyperactivity disorder (ADHD) participants have a threefold greater incidence of CI compared to the general population^47,48^. A working memory task assessed using functional MRI in adults with ADHD compared to age-matched controls supports differences in functional connectivity between groups in neural networks that include the cuneus^49^. Our results report two distinct regions of the cuneus correlating to the fast and slow fusional vergence systems that are significantly correlated to behavior. Perhaps, it is the intersection of visual attention and fusional vergence function residing within the cuneus that results in part from the oculomotor dysfunctions observed within CI.

### Clinical implications of research and future direction

The knowledge gained from this study is the critical first step toward understanding the neurophysiology of CI. From a clinical standpoint, CI is well understood as a condition in which the eyes have a tendency to deviate outward (exophoria at near), and the eyes' compensatory fusion ability is inadequate (positive fusional vergence)^50^. However, until now, there has been limited information, from just three studies with small sample sizes (N = 4 to N = 7), about how the neurology of vergence differs in BNC versus CI populations^17,28,31^. With the new knowledge gained from this study, future research can be planned to compare the effects of vision therapy protocols that target different vergence functions and determine the effects on the key cortical vergence areas as identified in this study, such as the cuneus. Our current results show that two distinct regions within the cuneus are significantly correlated with the fast and slow vergence systems. Significant differences are observed between BNC and CI groups for both functional activity and vergence behavior. Future studies investigating the intersection of oculomotor vergence function and visual attention would be prudent to further explore how new therapeutic interventions may be designed. Through these efforts, researchers will be able to develop and optimize novel therapeutic interventions that may further improve visual function for those with CI, as well as potentially reduce the amount of time needed to attain remediation. Such benefit has a broad impact on academic and professional success as well as recreational activities such as reading, sports, virtual reality gaming, and interfacing with electronic platforms, many of which are small hand-held devices held close to the eyes.

## Methods

### Design, participants, and clinical signs and symptoms

The Convergence Insufficiency Neuro-Mechanism in Adult Population Study (CINAPS) is a registered randomized clinical trial (NCT03593031 with ClinicalTrials.gov posted July 19, 2018) where all methods were carried out in accordance with the CONSORT agreement^51^. All participants signed written informed consent approved by the institutional review boards of New Jersey Institute of Technology and Rutgers University-Newark. All experimental protocols were approved by the institutional review boards of New Jersey Institute of Technology and Rutgers University-Newark. We enrolled 50 BNC and 50 symptomatic CI participants from 18 to 35 years of age and randomized each group to 12 h (two 1-h visits per week) of either office-based vergence/accommodative therapy or office-based placebo therapy^52^. We followed the protocols established in multiple randomized clinical trials which included a combination of office-based therapy with home therapy reinforement^13,15–17,23,53,54^. Thus, in addition to the office-based therapy, both groups were instructed to also perform 10 min of home-based therapy three times per week on the days they did not participate in office-based therapy. Home-based therapy was performed on a computer, and the amount of time spent was monitored by the research team to assess home compliance. Complete details of our randomized clinical trial design have been published^27^. A comprehensive oculomotor examination was administered to all eligible participants by an optometrist (co-author MS) who used the results to classify the participant as either BNC, symptomatic CI, or ineligible for study due to other vision or brain dysfunction or injury. We excluded any potential participant with amblyopia, anisometropia, vertical heterophoria, or reduced stereopsis. The criteria for symptomatic CI were consistent with previous randomized clinical trials^13,15–17,23,53^. Briefly, symptomatic CI was defined using the following criteria: (1) symptom score of ≥ 21 on the validated Convergence Insufficiency Symptom Survey (CISS)^55,56^; (2) reduced positive fusional vergence at near (a measure of the amount of prism required to disrupt binocular vision) either failing Sheard’s criterion^57^ or < 15 prism diopters (∆) base-out at near (40 cm); (3) receded near point of convergence (the closest point to which the eyes can converge, measured along the midline measured from the nasion) of ≥ 6 cm; and (4) an exophoria (outward deviation of the eye when binocular vision is disrupted) of at least 4∆ more exo at near (40 cm) compared to far (6 m). After completion of the 12 h of office-based therapy and 3 h of home-based therapy, the masked examiner (co-author MS) repeated the initial oculomotor test battery. The full description of the study design with inclusion and exclusion criteria, and details of the diagnostic criteria for symptomatic CI, BNC, masking, statistical power analysis, the binocular vision, and accommodative testing, and the office-based vergence accommodative therapy and placebo therapy protocols, are available in our prior publication^27^. Herein, we report the baseline data to compare the groups to identify potential neural mechanistic differences between BNC and CI participants.

### Behavioral experiments

#### Behavioral fast fusional vergence eye movements

The fast fusional disparity system was studied using symmetrical 4° (7∆) binocular step stimuli. While, several different step stimuli were studied to reduce anticipatory cues^29,58–60^, in the interest of brevity only the 4° step responses stimulated by an initial vergence angular demand of 6°, which then abruptly changed to a final vergence angular demand of 10°, were analyzed for this study. A haploscope presented disparity stimuli, utilizing fixed focal lengths to each eye (40 cm from the stimulus to the partially reflective mirror to participant’s globe) which kept accommodative cues constant at 2.5D. The stimulus was a Gabor patch similar to that used by other researchers^61^. Participants were carefully aligned so that visual stimuli were presented on their midline within a darkened room to reduce proximal cues. Eye movements were digitized at 240 frames per second using an ISCAN RK-826PCI (Burlington, MA, USA) (4 ms temporal resolution and 0.3° spatial resolution) that utilizes infrared light (940 nm). By synchronizing the eye tracker with the visual displays through our custom software^62^, we could perform detailed temporal analyses. Participants were properly refracted during all eye movement experiments. The direction (inward or outward) of the stimulus change, and the time at which the stimulus changed, were both randomized to reduce anticipatory cues^59^. Each eye movement recording was 4 s in duration, and the entire oculomotor experiment lasted 50 min. Eye movements were calibrated monocularly with six vergence angular demands over the range of the visual stimuli. Eye movement responses were denoted as degrees of angular rotation where convergence (inward rotation) was plotted as positive. Latency was measured as the time when the position trace from the initial vergence angle deviated by 5%. The final response amplitude was the average of the last half-second of the vergence eye movement response. The first derivative was computed as the velocity response, where the maximum value within the transient portion of the response was measured as peak velocity.

#### Behavioral slow fusional phoria adaptation

The slow fusional vergence system was assessed by comparing the near phoria measurement at baseline (with the participant’s habitual eyeglass prescription). The Modified Thorington technique, using the Bernell Muscle Imbalance Measure (MIM) card, a penlight, and a Maddox Rod (Bernell Corp., South Bend, IN, USA) was used to measure the near phoria^63^. The baseline phoria was recorded twice and averaged. Phoria adaptation disorder has been suggested to be an underlying mechanism in symptomatic CI patients^26^. To test this hypothesis, a lose 6Δ base-out wedge prism on a rod was placed in front of the participant’s right eye for the duration of the experiment after two baseline phoria measurements were recorded. The 6Δ wedge prism was chosen based on previous research showing that this magnitude maximizes the amount of recognizable change in phoria adaptation without creating retinal disparity that a CI participant would be unable to fuse^64^. This asymmetrical condition (prism only placed before one eye) was used because previous research demonstrated that this protocol provides a greater change in magnitude and rate of adaptation than a symmetrical condition^65^. To start the procedure, the right eye was occluded for 15 s, hence the vergence system was within an open loop state. As soon as the eye was uncovered, the participant was instructed to report the location of the red vertical line from the Maddox rod on the MIM card. The participant was then instructed to fixate binocularly on a single column of 20/30 letters from a fixation stick (Gulden Fixation Stick # 15302) for 30 s (with prism in front of right eye). Then, another phoria measurement was recorded with the vergence system being under open loop conditions. This measurement was repeated every 30 s over a period of 7 min, for a total of 15 measurements over the adaptation period similar to other studies^39,66^. The data were fit with an exponential decay function^66^. The magnitude was calculated as the change from the initial to the last phoria measure. The rate of phoria adaptation was calculated as the magnitude divided by the time constant measured from the exponential fit equation. The magnitude and rate were the primary metrics used to assess the slow fusional vergence system.

### Functional imaging experiments

#### Instrumentation and image acquisition parameters

A 3T Siemens TRIO with a 12-channel head coil (Siemens Medical Solutions, USA) from the Rutgers University Brain Imaging Center was utilized for all functional MRI experiments. An Eyelink-1000 system (SR Research, Canada) recorded the right-eye movements at 250 Hz to ensure that the participant performed the requested visual tasks and was calibrated before image acquisition using a 5-point calibration. A mirror on the headcoil, angled in front of the eyes, and situated 87.5 cm away from participant’s nasion, allowed the participant to view a screen that had 1920 by 1080 pixel resolution. A magnetization-prepared rapid acquisition gradient-echo (MP-RAGE) sequence was used to acquire a high-resolution anatomical reference with the following parameters: 1900 ms for time repetition (TR), 2.52 ms for time echo (TE), 900 ms for longitudinal relaxation time (T1), 9° flip angle, 256 mm field of view (FOV), 1.0 × 1.0 × 1.0 mm^3^ spatial resolution, and 175 slices within an axial orientation. Functional imaging experiments were acquired using an echo planar imaging (EPI) sequence with the following parameters: 2000 ms for TR, 13 ms for TE, 90° flip angle, 192 mm FOV, 3.0 × 3.0 × 3.0 mm^3^ spatial resolution, and 53 slices in an axial configuration. Participants who needed eyeglasses used fMRI compatible glasses to the closest 0.5D of their refractive prescription throughout the entire experimental session.

#### Visual stimuli for functional imaging experiments

The fast and slow fusional vergence systems were studied using functional imaging employing established methods published by our group using these exact protocols^67,68^. The visual stimuli are different compared to the behavioral studied performed in the laboratory because of the physical constraints of the magnet. For example, we could not easily place a prism in front of the eye while a participant was in the bore of the magnet. The fast fusional vergence experiment utilized a set of eccentric squares that stimulated 4° and 6° symmetrical disparity vergence jump step responses analogous to the behavioral experiment. The stimulus target extended 2° by 2°. Before the imaging experiment began, participants practiced free fusion (the ability to voluntarily converge or diverge their eyes to merge the two targets into one image) in the laboratory to ensure that they understood the directions and could perform the task. The timing protocol began with a “rest” block of sustained fixation for 21 s. Eight vergence step stimuli were then presented between 2 to 5 s per eye movement using the following order: 4° convergence (Con), 4° Con, 6° divergence (Div), 4° Con, 4° Con, 6° Div, 4° Con, and 4° Div. The vergence eye movement “task” block was 19 s in duration. The stimuli began with sustained fixation (21 s) and alternated with vergence step responses (19 s) for 5.5 cycles. There were 110 volumes (full brain images) collected or 220 s of image acquisition.

Within the same imaging session, the slow fusional system was studied using a sustained fixation task to adapt the phoria. A single square centered in the middle of the screen was presented to induce the perception of far distance (far disparity) viewed for 90 s followed by two eccentric squares that required the participant to converge and fuse, to stimulate near disparity viewed for 90 s, repeated three times for a total of 270 volumes or 540 s of image acquisition as shown in our prior study^68^. The time to adapt the phoria for 90 s was choosen because that was the amount of time observed from our behavioral studies where the largest change in phoria occurred given the exponential decay of the system. While longer periods of phoria adaptation may be desirable to study the slow fusional system, longer durations of phoria adaptation placed within a block design lead to long experiments where it is more likely for the participant to move and thus lead to data loss. The change in disparity was 6∆ base-out, which was analogous to the 6∆ base-out prism used in the behavioral experiment. Both the fast and slow fusional vergence fMRI experimental protocols were found to be repeatable by studying BNC participants to determine how well the visual stimuli protocol elicited reliable activation within the vergence neural substrates on different days^67,68^.

#### Imaging pre-processing

Both functional image datasets were analyzed with the MATLAB (Mathworks, MA, version 2015a) toolbox SPM 12 (Wellcome Centre for Human Neuroimaging, UCL, UK), using the default parameters. The following preprocessing protocol was conducted on each EPI functional image dataset for each participant for both the fast and slow fusional vergence experimental tasks. First, volumes were realigned to the initial volume, where head motion was reported for the following six directions: x, y, z, pitch, yaw, and roll movements between and within slices. Any data set with 1 mm or more of mean motion or more than 3 mm between samples were not further analyzed. Second, volumes were co-registered to each participant’s MP-RAGE anatomical dataset. Third, images were segmented into tissue-probability maps of cerebrospinal fluid (CSF) and white matter (WM) using a threshold of 97% or greater to extract a tissue type mask. The blood oxygen level-dependent (BOLD) fMRI time-series were extracted from the CSF and WM regions for each participant. The five principal components were calculated using a principal component analysis for CSF and WM time series^69^. Fourth, using each participant’s anatomical image as a reference, the BOLD fMRI images were transformed into the Montreal Neurological Institute (MNI) template (MNI152) and resampled to a 2 mm isotropic voxel-size using a 4th-degree b-spline interpolation with the SPM12 normalize function. A total of 34 nuisance variables (6 movements, 6 auto-regressions, 12 quadratics^70,71^, first 5 CSF principal components and the first 5 WM principal components) were regressed out from the BOLD fMRI data within the MNI standard space to reduce motion-related and physiological noise artifacts on task activation. Fifth, imaging datasets were high pass filtered with a cutoff frequency equal to 0.01 Hz for the fast fusional experimental and 0.0039 Hz for slow fusional experimental imaging datasets. Lastly, all images were spatially smoothed with a Gaussian kernel of 6 mm full width half maximum (fwhm).

#### Whole-brain activation maps

A general linear model (GLM) was used to calculate functional activity for each fusional experiment per participant^72^. Beta weight activation coefficients were calculated on a voxel-wise level. Each experiment (fast and slow fusional vergence) used a separate design matrix to model the hemodynamic BOLD response. For the fast fusional experiment, the design matrix was the sustained fixation resting blocks and the vergence eye movement task blocks convolved with a canonical waveform of a double gamma function. Hence, a contrast spatial map was generated comparing the vergence jump task blocks to the sustained fixation rest blocks. For the slow fusional experiment, since behavioral studies show that the phoria response exhibits an exponential decay, the GLM was calculated using a delta block of 4 s combined with the exponential decay obtained from the behavioral phoria adaptation experiment. The delta block was then convolved with a canonical double gamma response function. The functional imaging beta weights were calculated for the delta response using the GLM for each participant and the imaging data were displayed as axial and sagittal slices using AFNI^73^ or on a 3D model brain using BrainNetViewer^74^.

### Statistical analyses

#### Behavioral statistical analyses (clinical signs and symptoms, eye movements and phoria adaptation)

The following clinical measurements were compared between the BNC and CI groups using a two-sample unpaired t-test: near point of convergence, positive fusional vergence, difference of near and far phoria, and the CISS. For the fast-fusional experiment, a two-sample unpaired t-test compared the BNC and CI groups for the following parameters of the 4° symmetrical disparity vergence responses: latency, peak velocity, final amplitude, and the mean beta weights from an ROI. For the slow-fusional experiment of phoria adaptation, a two-sample unpaired t-test assessed differences between the BNC and CI groups for the magnitude and the rate of phoria adaptation and the mean beta weights from an ROI. For all behavioral parameters, the T value was calculated using SPSS Statistics (IBM, NY, version 20). The study was sufficiently powered^75^, and the data were normally distributed.

#### Functional imaging statistical analyses

For the group-level activation maps, a one-sample t-test was performed on the datasets. The resulting images were thresholded for significance using a voxel-level value of *p* < 0.001 and then corrected for multiple comparisons using the whole-brain, cluster-wise family-wise error (FWE) with a threshold of *p* < 0.05^76^. The group-level differences between the BNC and CI groups for the brain activation datasets were compared using a two-sample unpaired t-test for each voxel using an initial threshold of *p* < 0.001. Then, the data were corrected for multiple comparisons using FWE cluster-wise correction^76^ for *p* < 0.05 for both the fast and slow fusional imaging experiments.

#### Linear regression analyses between behavior and functional activity

Correlation between the brain activity beta weights and behavioral data were assessed using a few methods. For the first correlation method, the neural substrates showing significant differences between the groups were identified. The MNI coordinate of the most significant T value was extracted and a 5 mm radius sphere consisting of 81 voxels was created around this peak voxel coordinate using the MarsBaR MATLAB toolbox as part of SPM^77^. Our prior studies showed that the 5 mm radius sphere produced the highest intraclass correlation coefficients within our repeatability studies^67,68^ and has been used in a prior oculomotor fMRI investigation^78^. Additionally, the mean beta weights from 3 and 7 mm radius spheres consisting of 19 and 179 voxels, respectively were also computed for comparison to the 5 mm radius sphere results. The average beta weight from each sphere was computed. Then, for the fast fusional experiment, the averaged beta weights of the significantly different ROIs were correlated to the peak velocity and final amplitude of the vergence eye movements experimental results using a linear regression-based approach. For the slow fusional experiment, the beta weights of the significantly different ROIs were correlated with the following: (1) the magnitude of phoria adaptation, and (2) the rate of phoria adaptation. The last correlation method performed a voxel-wise correlation of the entire brain activation with vergence peak velocity and final amplitude for the fast fusional imaging dataset and with magnitude and rate of phoria adaptation for the slow fusional imaging dataset using SPM. The Pearson correlation coefficient with *p-*value was calculated using MATLAB for each correlation analysis.

## Supplementary Information

Supplementary Information.
