# Supplementary material for: Underlying neurological mechanisms associated with symptomatic convergence insufficiency
Source: Sci Rep. 2021 Mar 22;11:6545. doi: 10.1038/s41598-021-86171-9 (PMC7985149; doi:10.1038/s41598-021-86171-9)
Supplement: Supplementary file 1 — Supplementary Information. [file 41598_2021_86171_MOESM1_ESM.docx]

**Supplemental Material:**

Underlying neurological mechanisms associated with symptomatic convergence insufficiency

Tara L. Alvarez ([alvarez@njit.edu](mailto:alvarez@njit.edu) )^1*^, Mitchell Scheiman ([mscheiman@salus.edu](mailto:mscheiman@salus.edu) )^2^, Cristian Morales ([cm467@njit.edu](mailto:cm467@njit.edu) )^1^, Suril Gohel ([gohelsu@shp.rutgers.edu](mailto:gohelsu@shp.rutgers.edu) )^3^, Ayushi Sangoi ([aks84@njit.edu](mailto:aks84@njit.edu))^1^, Elio M. Santos ([esantos86@gmail.com](mailto:esantos86@gmail.com))^1^, Chang Yaramothu ([chang.yaramothu@njit.edu](mailto:chang.yaramothu@njit.edu) )^1^, John Vito d’Antonio-Bertagnolli ([john.vito@njit.edu](mailto:john.vito@njit.edu) )^1^, Xiaobo Li ([xiaobo.li@njit.edu](mailto:xiaobo.li@njit.edu) )^1^, Bharat B. Biswal ([bharat.biswal@njit.edu](mailto:bharat.biswal@njit.edu) )^1^

**Affiliations:**

^1^Biomedical Engineering, New Jersey Institute of Technology, Newark, NJ, USA

^2^Pennsylvania College of Optometry, Salus University, Philadelphia, PA, USA

^3^Department of Health Informatics, Rutgers University School of Health Professions, Newark, NJ, USA


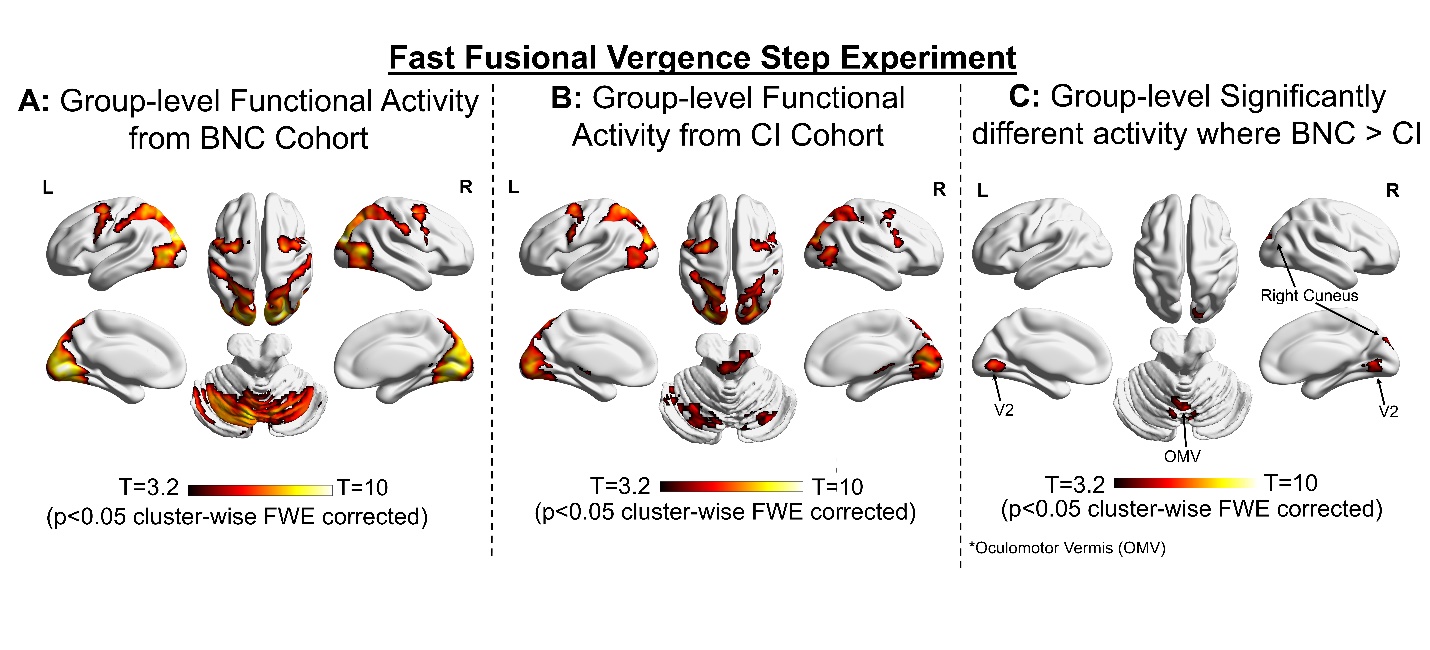


**Supplemental Figure 1:** Fast fusional vergence jump step experiment. Group-level one sample t-test of the sustained fixation rest block compared to task block comprise of 8 vergence eye movements for the BNC (**A**) and CI (**B**) cohorts with an unpaired two sample t-test comparing which ROIs were significantly greater in BNC compared to CI cohorts (**C**)


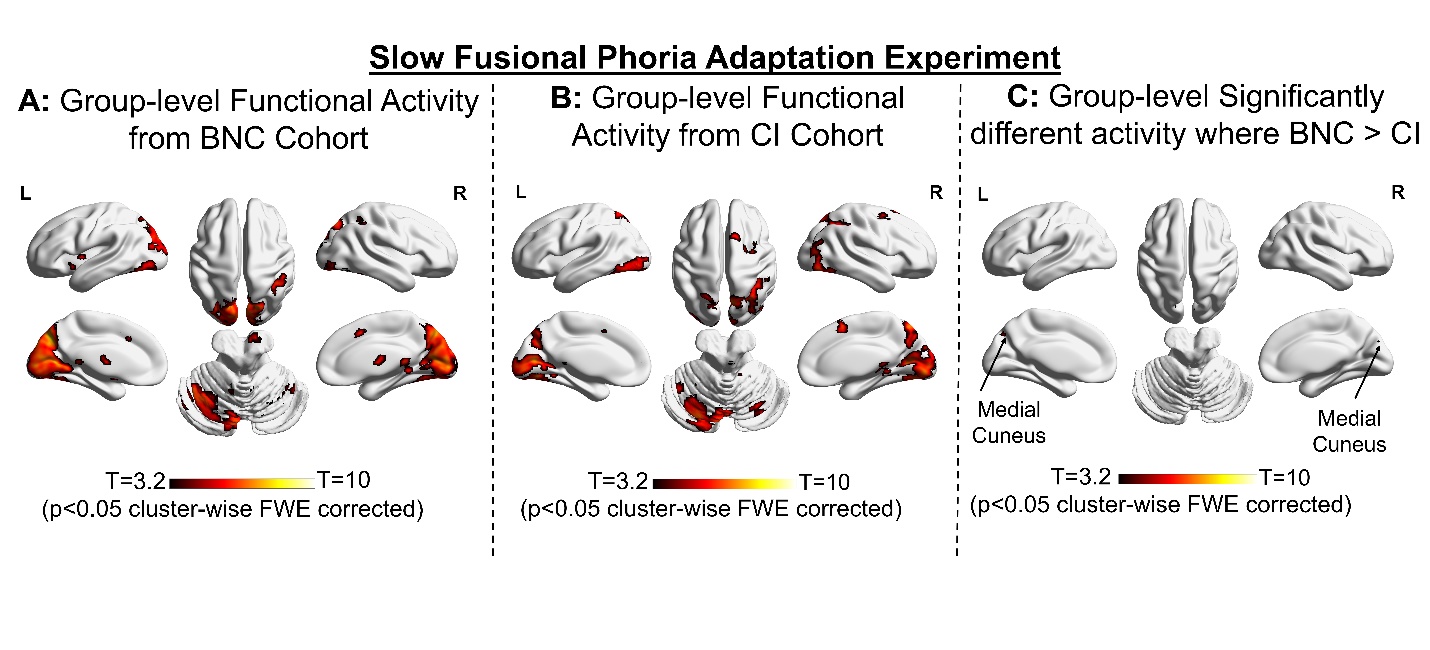


**Supplemental Figure 2:** Slow fusional phoria adaptation experiment. Group-level one-sample t-test of the sustained fixation that alternated between near and far vergence demand to stimulate the visual demand of 6∆ base-out prism for the BNC (**A**) and CI (**B**). An unpaired t-test comparing which ROIs were significantly greater in BNC compared to CI cohorts (**C**)
